# Disruption of A2AR-D2R Heteroreceptor Complexes After A2AR Transmembrane 5 Peptide Administration Enhances Cocaine Self-Administration in Rats

**DOI:** 10.1007/s12035-018-0887-1

**Published:** 2018-01-30

**Authors:** Dasiel O. Borroto-Escuela, Karolina Wydra, Xiang Li, David Rodriguez, Jens Carlsson, Joanna Jastrzębska, Malgorzata Filip, Kjell Fuxe

**Affiliations:** 1 Department of Neuroscience, Karolinska Institutet, Retzius väg 8, 17177 Stockholm, Sweden; 2 Department of Biomolecular Science, Section of Physiology, University of Urbino, Campus Scientifico Enrico Mattei, via Ca’ le Suore 2, 61029 Urbino, Italy; 3 Observatorio Cubano de Neurociencias, Grupo Bohío-Estudio, Zayas 50, 62100 Yaguajay, Cuba; 4 Institute of Pharmacology, Department of Drug Addiction Pharmacology, Polish Academy of Sciences, 12 Smetna Street, 31-343 Kraków, Poland; 5 College of Life Sciences, Jilin University, Qianjin Street No. 2699, Changchun, 130012 China; 6 Science for Life Laboratory, Department of Biochemistry and Biophysics, Stockholm University, SE-106 91 Stockholm, Sweden; 7 Science for Life Laboratory, Department of Cell and Molecular Biology, Uppsala University BMC, Box 596, 751 24 Uppsala, Sweden

**Keywords:** Adenosine A2A receptor, Cocaine self-administration, Dopamine D2 receptor, Heteroreceptor complexes, Interfering peptides, Dimerization, Substance use disorder

## Abstract

Antagonistic allosteric A2AR-D2R receptor-receptor interactions in heteroreceptor complexes counteract cocaine self-administration and cocaine seeking in rats as seen in biochemical and behavioral experiments. It was shown that the human A2AR transmembrane five (TM5) was part of the interface of the human A2AR-D2R receptor heteromer. In the current paper, the rat A2AR synthetic TM5 (synthTM5) peptide disrupts the A2AR-D2R heteroreceptor complex in HEK293 cells as shown by the bioluminescence resonance energy transfer method. Rat A2AR synthTM5 peptide, microinjected into the nucleus accumbens, produced a complete counteraction of the inhibitory effects of the A2AR agonist CGS21680 on cocaine self-administration. It was linked to a disappearance of the accumbal A2AR-D2R heteroreceptor complexes and the A2AR agonist induced inhibition of D2R recognition using proximity ligation assay and biochemical binding techniques. However, possible effects of the A2AR synthTM5 peptide on accumbal A2AR-D3R and A2AR-D4R heteroreceptor complexes remain to be excluded. Evidence is provided that accumbal A2AR-D2R-like heteroreceptor complexes with their antagonistic receptor-receptor interactions can be major targets for treatment of cocaine use disorder.

**Electronic supplementary material:**

The online version of this article (10.1007/s12035-018-0887-1) contains supplementary material, which is available to authorized users.

## Introduction

Adenosine 2A receptor (A2AR)-dopamine D2 receptor (D2R) heteroreceptor complexes in the brain may offer novel targets for treatment of cocaine use disorder based on the fact that a well-established antagonistic allosteric receptor-receptor interactions exist in the A2AR-D2R heteroreceptor complexes [1–4]. These heteroreceptor complexes are located in the ventral striato-pallidal GABA neurons, representing an anti-reward system [5–7]. The important role of D2R in cocaine use disorder was demonstrated by the findings of persistent elevations of the high affinity type D2R upon chronic cocaine self-administration [8]. Furthermore, D2R activation produces strong relapse of cocaine-seeking in rats [9, 10]. There is evidence that antagonistic allosteric A2AR-D2R receptor-receptor interactions in heteroreceptor complexes counteract cocaine self-administration and cocaine seeking in animals as seen both at the biochemical and behavioral level [9, 11–16].

Our recent observations show that cocaine self-administration selectively activates the antagonistic A2AR-D2R receptor-receptor interaction in the ventral striatum, but not in the dorsal striatum [17]. These results indicate that the A2AR-D2R heteroreceptor complexes in the nucleus accumbens mediate the anti-cocaine actions of A2AR agonists on cocaine reward. Further evidence for this view was obtained newly by the demonstration with using the proximity ligation assay that cocaine self-administration specifically increases the A2AR-D2R and D2-sigma1R heteroreceptor complexes in the nucleus accumbens shell vs the dorsal striatum [18]. The results also open up the possibility that increased numbers of the A2AR-D2R-sigma1R heteroreceptor complexes can be formed in the nucleus accumbens shell upon cocaine self-administration with enhanced antagonistic allosteric A2AR-D2R receptor-receptor interactions [6, 18–20].

In 2010, an atomic resolution model of the human A2AR-D2R heteromer was achieved [21]. It was shown that the human A2AR TM5 may be part of the interface of the human A2AR-D2R heteromer. Therefore, in the current paper, we tested the effects of the rat A2AR synthetic TM5 (synthTM5) peptide, microinjected in the nucleus accumbens, on cocaine self-administration. Also, its effects on the densities of the A2AR-D2R heterorecepor complexes and their allosteric receptor-receptor interactions were studied in the nucleus accumbens of the rat brain. For specificity reasons, the dorsal striatum was also studied. The effects of the rat A2AR synthTM5 peptide were characterized in cellular models by studying its interference with the BRET^1^ signal from the A2AR-D2R heteromer. Again for specificity reasons also, the rat A2AR synthTM2 peptide was tested [21]. The results give evidence that the disruption of the accumbens A2AR-D2R heteroreceptor complex by the A2AR synthTM5 peptide blocks the inhibitory action of the A2AR agonist on cocaine self-administration and restores cocaine reward in rats. The A2AR-D2R heteromer becomes a novel target in treatment of cocaine use disorder.

## Materials and Methods

### Animals

Male Sprague–Dawley (derived from the licensed animal breeder Charles River, Sulzfeld, Germany), weighing between 260 and 310 g at the beginning of the experiment, were used. The animals were housed individually in standard plastic rodent cages (39 cm × 28 cm × 28 cm) in a colony room maintained at 21 ± 1 °C and 45–65% humidity under a 12-h light-dark cycle (lights on at 6:00 am). Rodent food (VRF1 pellets, UK) and water were available ad libitum except for the period of the initial training sessions when rats were maintained on limited water. All protocols were conducted during the light phase of the light-dark cycle between 9:00 and 13:00 h. All animals used in the study were experimentally naive. The experiments were carried out in accordance with the European Directive 2010/63/EU and were approved by the Ethical Committee at the Institute of Pharmacology, Polish Academy of Sciences, Krakow.

### Drugs

Cocaine hydrochloride (Sigma-Aldrich; St. Louis, USA) was dissolved in sterile 0.9% NaCl and administered i.v. in a volume of 0.1 ml per infusion. CGS 21680 (Tocris, UK, 0.1 mg/kg) was dissolved in 0.9% NaCl and administrated i.p. 10 min before 2-h self-administration session in a volume of 0.1 ml/kg. Dose of A2AR agonist was established based on previous behavioral studies [9]. Vehicle (Krebs-Ringer; in mM: NaCl 120, KCl 2, MgCl_2_ 1.8, CaCl_2_ 1.2, Na_2_SO_4_ 0.5, NaHCO_3_ 20, KH_2_PO_4_ 0.5, D-glucose 6.8, pH 7.4) or the synthTM5 peptide diluted in the vehicle solution were injected into nucleus accumbens at a constant flow rate (0.5 μl, infused during 1 min) on each side.

### Synthetic Transmembrane Peptides

The two synthetic peptides corresponding to the TM2 (synthTM2: KKKFFVVSLAAADIAVGVLAIPFAITIKKK) and TM5 (synthTM5: KKKMNYMVYYNFFAFVLLPLLLMLAIYLKKK) helices of the rat A2AR were purchased from VTG (Sweden) or CASLO (Denmark). At both the N- and C-terminal juxtamembrane sequence of the rat A2AR synthTM2 and synthTM5 petides, the tribasic sequence lysine (KKK) was introduced, found in many membrane proteins to ensure incorporation into the cell plasma membrane. The synthTM5 peptide (0.1 μM, diluted in the vehicle solution) was administered into nucleus accumbens twice (22 h and 20 min) before last cocaine self-administration session in a volume 0.5 μl infused during 1 min on each side. The amount given each time was 0.05 pmol leading to a total amount of 0.1 pmol per side.

### Surgery and Behavioral Experiments

Behavioral experiments included acquisition and maintenance of intravenous cocaine self-administration.

### Surgery

Eighteen-hour water-restricted rats were trained to press a lever for 2 h daily in standard operant chambers for 5 days (Med-Associates, USA) under a fixed ratio 5 (FR-5) schedule of water reinforcement. Two days following lever press training and free access to food and water, animals were anesthetized with ketamine HCl (75 mg/kg, *i.m.*; Biowet, Poland) and xylazine (5 mg/kg, *i.m.*; Biowet, Poland) cocktail and chronically implanted with a silastic catheter in the external jugular vein, as described previously [12]. Immediately after the catheter implantation, rats were stereotaxically implanted with stainless steel guide cannulae (22-gauge, 10 mm long; Plastic One, USA), one on each side of the nucleus accumbens. Guide cannulae were implanted into the nucleus accumbens shell at the following coordinates from the Bregma: anteroposterior (AP) = 1.7 mm; mediolateral (ML) = ± 0.75 mm; and dorsoventral (DV) = − 6 mm, according to the atlas of Paxinos and Watson [22]. The guide cannulae were affixed to the skull with two miniature stainless steel screws and dental acrylic cement.

Internal cannulae (28-gauge, 12-mm length, PlasticsOne, USA) were inserted into the guide cannulae (internal cannulae extending 2 mm beyond the end of guide cannulae) after obturator removal. The microinjection unit was organized from a polyethylene tube (OD D 0.023, ID 0.041, PlasticOne, USA) connected to two 1-ml Hamilton syringes on one end and to bilateral injection canualae on the other end. The microinjection volume of 0.5 μl was delivered bilaterally over 1 min by the microinjection pump (CMA/Microdialysis, Dalvägen, Sweden). For details on volume and amounts of synthTM5 peptide and of vehicle microinjected, see part on synthetic transmembrane peptides.

Rats were allowed 10 days to recover from surgery before the start of the experiments. Catheters were flushed daily with 0.2 ml of saline solution containing heparin (100 U/ml, Biochemie, Austria) and 0.1 ml of a cephazolin solution (100 mg/ml Biochemie GmbH, Austria) to prevent catheter non-patency. Catheter patency was tested periodically with the short-acting barbiturate anesthetic methohexital (10 mg/kg, *i.v*.), which induced the loss of consciousness within 5 s. No problems with catheter patency were reported in the tested rats.

### Cocaine Self-Administration

After the recovery period, all animals began lever pressing for cocaine reinforcement during 2-h daily sessions performed 6 days per week. The house light was illuminated throughout each session. Each press on the “active” lever (FR-5 schedule of reinforcement) resulted in a 5-s infusion of cocaine (0.5 mg/kg per 0.1 ml) and a 5-s presentation of a stimulus complex (activation of the white stimulus light directly above the ‘active’ lever and the tone generator).

Following each injection, there was a 20-s time-out period during which responding was recorded, but had no programmed consequences. Presses on the ‘inactive’ lever were recorded, but not reinforced. After the 7 days of acquisition, rats were used to complete a cocaine (0.25–0.5 mg/kg/infusion) dose–response curve. Cocaine self-administration was conducted daily for 15 sessions. Following stabilization of responding rates with cocaine (0.25 mg/kg/infusion) self-administration, the animals were divided into separate groups (*n* = 6–7) to undergo test procedures. Vehicle or synthTM5 peptide (0.1 μM) was administered into nucleus accumbens twice (22 h and 20 min) before the last cocaine self-administration session as indicated above. CGS 21680 (0.1 mg/kg) was administrated 10 min before last cocaine self-administration session. Immediately after the last 2-h cocaine self-administration session, animals were either sacrificed (for biochemical experiments) or injected with pentobarbital and perfused intra-cardially (for IHC and in situ PLA experiments).

### Biophysical (BRET) Experiments

#### Plasmid Constructs, Cell Culture, and Transfection

Plasmid constructs were made using standard molecular biology techniques by employing PCR and fragment replacement strategies. For more details, see references [23–25].

#### BRET^1^ Saturation Assays

In the BRET^1^ assays, 48 h after transfection, HEK293T cells transiently transfected with a constant (1 μg) or increasing amounts (0.5–8 μg) of plasmids encoding for D2R^Rluc^ and A2AR^YFP^ were rapidly washed twice in PBS, detached, and resuspended in the same buffer. Cell suspensions (20 μg proteins) were distributed in duplicate into the 96-well microplates: black plate with transparent bottom (Corning 3651, Corning, Stockholm, Sweden) for fluorescence measurement or white plates with white bottom (Corning 3600) for BRET determination. For BRET^1^ ratio measurement, coelenterazine *h* substrate (Molecular Probes, Eugene, OR, USA) was added at a final concentration of 5 μM. Readings were performed 1 min after, and the BRET signal was detected using the POLARstar Optima plate reader (BMG Labtechnologies, Offenburg, Germany) that allows the sequential integration of the signals detected with two filter settings. The specificities of A2AR-D2R interactions were assessed by comparison with co-expression of D2R^Rluc^ and T2R20^YFP^. To determine the effects of the TM peptides, transfected HEK293T cells were incubated with 0.1 μM of the TM peptides at 37 °C for 2 h prior to performing BRET^1^ analysis. Data were then represented as a normalized BRET^1^ ratio, which was defined as the BRET ratio for co-expressed Rluc and YFP constructs normalized against the BRET ratio for the Rluc expression construct alone in the same experiment: BRET^1^ ratio = [(YFP emission at 530 ± 10 nm)/(Rluc emission 485 ± 10 nm)]–cf. The correction factor, cf., corresponds to (emission at 530 ± 10 nm)/(emission at 485 ± 10 nm) found with the receptor-Rluc construct expressed alone in the same experiment. BRET isotherms were fitted using a nonlinear regression equation assuming a single binding site, which provided BRET_max_ and BRET_50_ values as described previously [4].

#### BRET^1^ Competition Assay

HEK293T cells transiently co-transfected with constant amounts (1 μg) of plasmids encoding for D2R^Rluc^ and A2AR^YFP^ were incubated with the TM peptides at 37 °C for 2 h prior to performing BRET^1^ analysis. For BRET^1^ ratio measurement, coelenterazine *h* substrate (Molecular Probes, Eugene, OR, USA) was added at a final concentration of 5 μM, and readings were performed 1 min after. The BRET signal was detected as described above.

### Biochemical Radioligand Binding Experiments

#### Membrane Preparation

After cocaine self-administration, the rats were decapitated. The dorsal and ventral striatum were dissected out and immediately frozen on dry ice and stored at − 80 °C. Frozen rat striatum was homogenized in 5-ml ice-cold preparation buffer using a sonicator (Soniprep 150). The buffer contained 50 mM Tris-HCl, pH 7.4, 7 mM MgCl_2_, 1 mM EDTA, and a cocktail of protease inhibitors (Roche Diagnostics, Mannheim, Germany). The membranes were precipitated by centrifugation at 4 °C for 40 min at 40,000×*g* (Thermo scientific, Sorvall Lynx 6000, Stockholm, Sweden) and washed through rehomogenization in the same buffer once more. The protein concentration was determined for the membrane homogenates by means of BCA Protein Assay (Pierce, Sweden) using as a standard bovine serum albumin (BSA). Pelleted membranes were resuspended to a concentration of 0.4 mg/ml, immediately used or stored at − 80 °C until required.

#### [^3^H]-Raclopride Binding Experiments

Competition experiments of quinpirole (0.3 nM–3 mM) versus the D2-likeR antagonist [^3^H]-raclopride (2 nM; specific activity 78.1 Ci/mmol, PerkinElmer Life Sciences, Stockholm, Sweden) were carried out by membrane (20 μg per well) incubation at 30 °C for 90 min, in the presence or absence of 100 nM of the A2AR agonist CGS 21680. Nonspecific binding was defined by radioligand binding in the presence of 10 μM (+)-butaclamol (Sigma-Aldrich, Stockholm, Sweden). The incubation was terminated by rapid filtration through Hydrophilic (LPB) Durapore ®Membrane (Millipore, Stockholm, Sweden) using a MultiScreen™ Vacuum Manifold 96-well (Millipore Corp, Bedford, MA), followed by five washes (200 μl per wash) with ice-cold washing buffer (50 mM Tris-HCl pH 7.4). The filters were dried, 4 ml of scintillation cocktail was added, and the bound ligand was determined after 12 h by liquid scintillation spectrometry.

### Immunohistochemistry and In Situ PLA Experiments

#### Tissue Preparation

For immunohistochemical staining, the animals were immediately after the experimental sessions injected with pentobarbital (133.3 mg/kg, i.p; Biowet, Puławy, Poland) and perfused intracardially with saline followed by 4% paraformaldehyde solution (VWR, Gdańsk, Polska). Each brain was immersed in the same fixative for 12 h. The brain was left at 4–8 °C in 10% *w*/*v* sucrose up to 7 days followed by 30% *w*/*v* sucrose for 2 weeks.

#### In Situ Proximity Ligation Assay (In Situ PLA)

To study the effects of the synthTM5 peptide on the A2AR-D2R heteroreceptor complex density changes after cocaine self-administration, the in situ PLA was performed as described previously [4, 7, 26]. Free-floating formalin-fixed brain sections (30 μm-thick, cut using a cryostat) at Bregma level (1.0 mm) (see Online Resource 1) from rats after cocaine-self administration were employed using the following primary antibodies: rabbit monoclonal anti-D2R (HPA015691, 1 μg/ml, Human Atlas Project, Stockholm, Sweden) and rabbit monoclonal anti-A2AR (AB1559F, 1:250; Millipore, Sweden). Control experiments employed only one primary antibody or cells transfected with cDNAs encoding only one type of receptor. The PLA signal was visualized and quantified by using a Leica TCS-SL SP5 confocal microscope (Leica, USA) and the Duolink Image Tool software. Briefly, fixed free-floating rat brain sections (storage at − 20 °C in Hoffman solution) were washed four times with PBS and quenched with 10 mM glycine buffer, for 20 min at room temperature. Then, after three PBS washes, incubation took place with a permeabilization buffer (10% fetal bovine serum (FBS) and 0.5% Triton X-100 or Tween 20 in Tris buffer saline (TBS), pH 7.4) for 30 min at room temperature. Again, the sections were washed twice, 5 min each, with PBS at room temperature and incubated with the blocking buffer (0.2% BSA in PBS) for 30 min at room temperature. The brain sections were then incubated with the primary antibodies diluted in a suitable concentration in the blocking solution for 1–2 h at 37 °C or at 4 °C overnight. The day after, the sections were washed twice, and the proximity probe mixture (minus and plus probes, for details, see: Duolink instructions) was applied to the sample and incubated for 1 h at 37 °C in a humidity chamber. The unbound proximity probes were removed by washing the slides twice, 5 min each time, with blocking solution at room temperature under gentle agitation. The sections were incubated with the hybridization-ligation solution (BSA (250 g/ml), T4 DNA ligase (final concentration of 0.05 U/μl), Tween-20 (0.05%), NaCl 250 mM, ATP 1 mM, and the circularization or connector oligonucleotides (125–250 nM)) and incubated in a humidity chamber at 37 °C for 30 min. The excess of connector oligonucleotides was removed by washing twice, for 5 min each, with the washing buffer A (Sigma-Aldrich, Duolink Buffer A (8.8 g NaCl, 1.2 g Tris Base, 0.5 ml Tween 20, dissolved in 800 ml high purity water, pH to 7.4) at room temperature under gentle agitation and the rolling circle amplification buffer was added to the sections and incubated in a humidity chamber for 100 min at 37 °C. Then, the sections were incubated with the detection solution through hybridization (fluorescent oligonucleotide probes) in a humidity chamber at 37 °C for 30 min. In a last step, the sections were washed twice in the dark, for 10 min each, with the washing buffer B (Sigma-Aldrich, Duolink Buffer B (5.84 g NaCl, 4.24 g Tris Base, 26.0 g Tris-HCl, dissolved in 500 ml high purity water, pH 7.5) at room temperature under gentle agitation. The free-floating sections were put on a microscope slide, and a drop of appropriate mounting medium containing DAPI giving a blue staining of the nuclei (e.g., VectaShield or Dako) was applied. The cover slip was placed on the section and sealed with nail polish. The sections were protected against light and stored for several days at − 20 °C before confocal microscope analysis.

### Statistical Analysis

Data were analyzed using GraphPad Prism 5.0 (GraphPad Software Inc., San Diego, CA). All the data are shown as means ± SEM. In behavioral experiments, the number of responses on the active and inactive lever or the number of infusions was analyzed using a one-way analysis of variance (ANOVA) for repeated measurements, the latter analysis followed by post hoc Dunnett test. Instead, data from in situ PLA experiments showing cluster density (clusters per nucleus per sampled field) were analyzed using a one-way ANOVA followed by post hoc Tukey’s test. Data from the competition experiments were analyzed by nonlinear regression analysis. The effects of CGS 21680 on the K_iH_ and K_iL_values and on the proportion of D2R agonist binding sites in the high affinity state with or without A2AR synthTM5 peptide treatment were evaluated with paired Student’s *t* test. The percent change induced by CGS 21680 on the Ki_H_ and Ki_L_ values and in the proportion of D2-likeR in rats self-administering cocaine was compared using nonparametric Mann-Whitney *U* test. The number of rats (*n*) in each experimental condition is indicated in figure legends. The *P* value 0.05 and lower was considered significant.

## Results

### In Vitro Experiments on A2AR and D2R cDNA Cotransfected HEK293 Cells—Effects of the A2AR SynthTM5 Peptide

**Fig. 1:**
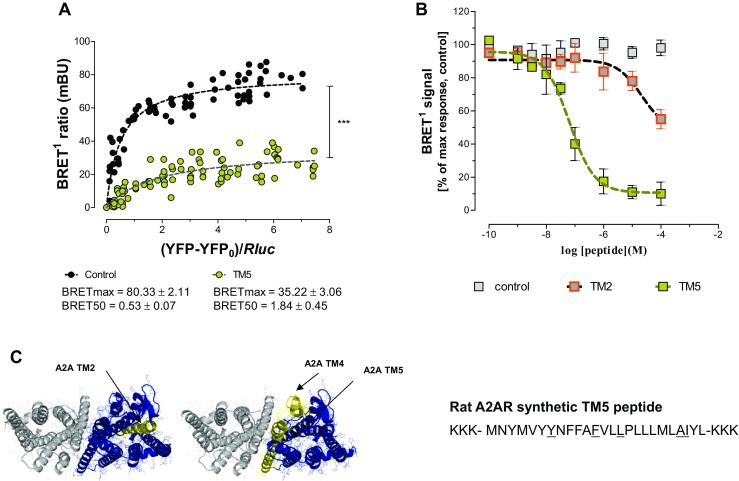
The effects of the rat A2AR synthTM5 peptide are shown on the A2AR-D2R heteromers in HEK293 cells using BRET^1^ saturation analysis (**a**) and BRET^1^ competition analysis (**b**). A highly significant reduction of the BRETmax value is found (***p < 0.001; Student’s *t* test; 10 experiments and 8 replicates). In the right panel, the high affinity and effective competition by the rat A2AR synthTM5 peptide for the D2R protomer is shown by the marked disappearance of the BRET^1^ signal at low concentrations. In contrast, the rat A2AR synthTM2 peptide produced only a weak effect at high concentrations. Mean ± SEM for each point is given (10 experiments and 8 replicates). **c** A molecular model is shown of the A2AR-D2R heterodimer to illustrate the presence of A2AR synthTM5 peptide in the interface, while A2AR synthTM2 is not part of the interface (the PDB coordinate of this molecular model was obtained from Borroto-Escuela et al. [27])

It was indicated that A2AR TM5 helix was involved in the human A2AR-D2R heterodimer interface [21] as illustrated by the molecular model in Fig. 1c. The human A2AR synthTM5 peptide may therefore reduce A2AR-D2R heteromerization by competition with the A2AR for binding to the interface with the D2R. Here, it was tested if a rat A2AR syntheticTM5 peptide (Fig. 1c) had such an ability using a quantitative BRET^1^ assay.

### BRET^1^ Saturation Analysis

As seen in Fig. 1a, the rat A2AR synthTM5 peptide at 1 μM produced a marked and highly significant reduction of the BRET^1^ signal with a reduction of the Bmax value and an increase in the BRET50 value.

### BRET^1^ Competition Analysis

It was found in concentration-response experiments (Fig. 1b) that the rat A2AR synthTM5 peptide produced a concentration-dependent inhibition of the BRET^1^ signal with an almost complete blockade of the BRET^1^ signal at 1–10 μM. In contrast, the human A2AR synth TM2 peptide which is not part of the A2AR-D2R receptor interface [21] only caused a modest disappearance of the BRET^1^ signal at 100 μM.

### In Vivo Experiments with the Rat A2AR SynthTM5 Peptide—Effects of Intra Accumbal Microinjections of the Peptide on Cocaine Self-Administration

#### Behavioral Analysis

**Fig. 2:**
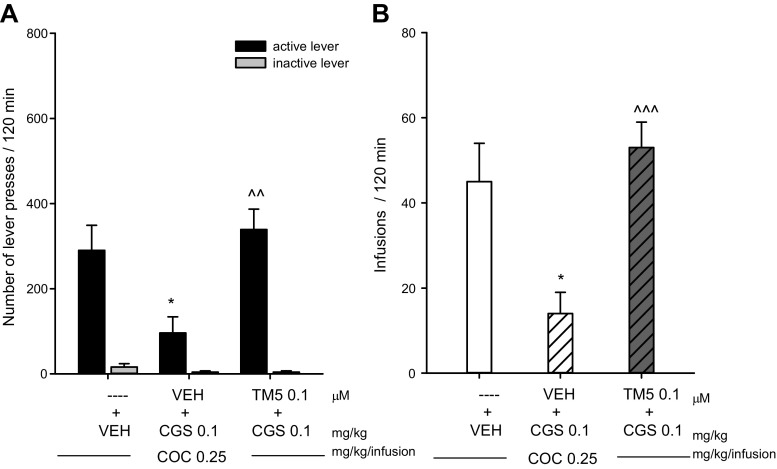
Intra-accumbal microinjections of the A2AR synthTM5 peptide (0.1 μM) (TM5) or vehicle (VEH) on the inhibitory effects of the A2AR agonist CGS 21680 (0.1 mg/kg; i.p.) during cocaine (COC; 0.25 mg/kg/infusion) self-administration. The number of active and inactive lever presses (**a**) and the number of cocaine infusions (**b**) are shown. Each bar represents the mean ± SEM. Number of rats = 6–7 per group. *P < 0.01 vs VEH + coc 0.25, active lever, ^^*P* < 0.01, ^^^*P* < 0.001 vs VEH + CGS 0.1 + coc 0.25, active lever. The vehicle and the rat synthTM5 peptide (0.1 μM, diluted in the vehicle solution) were administered into nucleus accumbens twice (22 h and 20 min) before last cocaine self-administration session in a volume 0.5 μl, infused during 1 min on each side. The amount given each time was 0.05 pmol leading to a total amount of 0.1 pmol per side

Following 14–16 sessions, rats acquired cocaine self-administration (i.e., they received > 25 infusions/2 h session) and displayed < 10% variation in the number of infusions received over the last 6 self-administration sessions. The mean number of cocaine infusions per day during the last 6 self-administration days varied from 25 to 34. The total cocaine intake was 202 ± 15 mg/rat (means ± SEM). Rats pressed significantly more on the active lever than on the inactive lever (*P* < 0.01) (Fig. 2a).

Immediately after 14 days of cocaine self-administration (22 h before the test), the rats were microinjected into the nucleus accumbens with vehicle or the rat A2AR synthTM5 peptide (0.1 μM, 0.5 μl infused during 1 min on each side). On the last day of cocaine (0.25 mg/kg/infusion) self-administration, the rats received a second intra-accumbal infusion with vehicle or synthTM5 peptide (0.1 μM, 0.5 μl infused during 1 min on each side) before injection with the A2AR agonist CGS 21680 (0.1 mg/kg) and the cocaine self-administration session.

CGS 21680 (0.1 mg/kg) in vehicle-treated rats significantly decreased the number of active lever presses [F(1, 11) = 6.986, *p* < 0.02] and the number of cocaine infusions [F(1, 11) = 8.00, *p* < 0.016] but did not alter the number of inactive lever presses [F(1, 11) = 1.844, *p* < 0.202] (Fig. 2a, b). The A2AR TM5 peptide significantly counteracted the CGS 21680 (0.1 mg/kg)-induced reduction in the number of active lever responses [F(1, 11) = 15.08, *p* < 0.002] and in the number of cocaine infusions [F(1, 11) = 27.61, *p* < 0.0002] (Fig. 2a, b). However, it did not change the number of inactive lever presses [F(1, 11) = 0.026, *p* = 0.873].

#### Neurochemical Analysis

##### 3H–Raclopride/Quinpirole Competition Binding Experiments

**Fig. 3:**
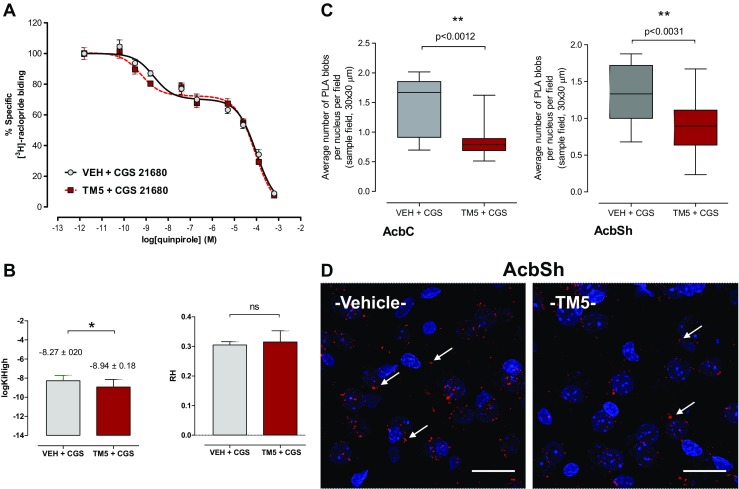
Ventral striatum. Effects of intra-accumbal microinjections of the rat A2AR synthTM5 peptide or vehicle in the presence of CGS 21680 during cocaine self-administration (see text to Fig. 2) on the ^3^H–Raclopride binding/quinpirole competition curves (**a**, **b**) and on the density of the PLA positive A2AR-D2R heteroreceptor complexes in nucleus accumbens core (AcbC) and nucleus accumbens shell (AcbSh) (**c**, **d**). The A2AR synthTM5 peptide microinjection produced a shift to the left of the D2R agonist induced competition curve in the high affinity but not in the low affinity range. This resulted in a significant reduction (*p* < 0.05) of the log K_iH_ value vs the VEH group (Student’s *t* test). Number of rats = 5 per group. **c** The microinjection of the A2AR synthTM5 peptide is shown to produce a significant reduction in the density of the PLA positive complexes per nucleus per cell in the AcbC and AcbSh (** *p* < 0.0012 and *p* < 0.0031, respectively). For sampling fields at Bregma 1.00 mm, see Online Resource 1. Mean ± SEM, number of rats = 5 per group. Student’s *t* test with Bonferoni correction. In the **d** panel, one representative example is given for the reduction of the densities of the red PLA positive A2AR-D2R heteroreceptor complexes after the rat A2AR synthTM5 peptide treatment vs vehicle treatment. Arrows point to some of the red PLA positive clusters. The length of the bar is 30 μm

Ventral striatum. The effects of the in vivo treatment with the A2AR agonist CGS 21680 were studied on the ability of the D2-likeR agonist quinpirole to compete with the D2-likeR antagonist 3H–Raclopride for the D2-likeR binding sites after the microinjections of A2AR synthTM5 peptide or vehicle into the nucleus accumbens. The log KiH values were found to be significantly reduced after treatment with the A2AR synthTM5 peptide vs vehicle in the ventral striatum (Fig. 3a, b) demonstrating an increase in the affinity of the high affinity D2-likeR agonist binding sites. The proportion of the D2-likeR in the high affinity state was unaffected (Fig. 3b). The competition curves showed that the affinity of the D2-likeR in the low affinity state was unaffected by the microinjection of the A2AR synthTM5 peptide into the nucleus accumbens.

**Fig. 4:**
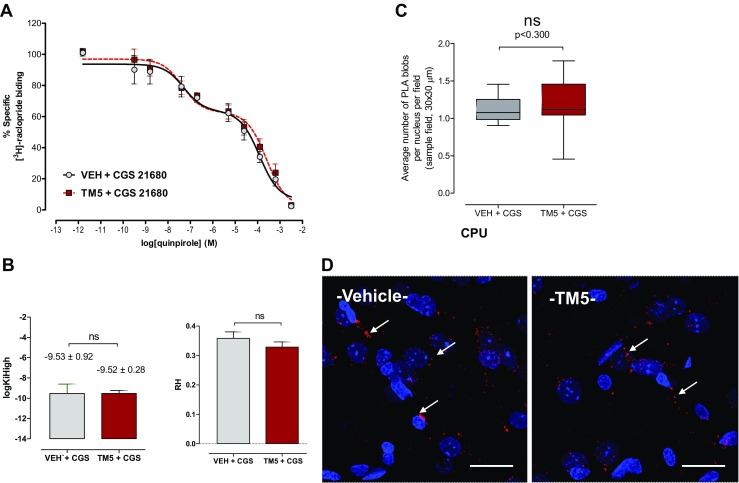
Control area (dorsal striatum). Effects of intra-accumbal microinjections of the rat A2AR synthTM5 peptide or vehicle in the presence of CGS 21680 during cocaine self-administration (see text to Fig. 2) on the 3H–Raclopride/quinpirole competition curves (**a**) and on the density of the PLA positive A2AR-D2R heteroreceptor complexes in the caudate putamen (**c**, **d**). For sampling fields at Bregma 1.00 mm, see Online resource 1. As seen in **a**, **b**, the A2AR synthTM5 peptide microinjection did not produce a change in the D2R agonist induced competition curve in the high affinity range nor in the low affinity range. In line with these results, the log K_iH_ values in the caudate putamen were not significantly altered by the A2AR synthTM5 peptide microinjection into the nucleus accumbens. **c** The intra-accumbal microinjection of A2AR TM5 peptide did not produce a significant change in the density of the PLA positive complexes per nucleus per cell vs vehicle treatment. Mean ± SEM, number of rats = 5 per group. Student’s *t* test with Bonferoni correction. In **d**, one representative example is given for the lack of changes in the densities of the red PLA positive A2AR-D2R heteroreceptor complexes after the A2AR TM5 peptide treatment vs vehicle treatment. Arrows point to some of the red PLA positive clusters. The length of the bar is 30 μm

Dorsal striatum (control area). In contrast to the ventral striatum, the 3H–Raclopride/Quinpirole competition curves were unaffected by the microinjection of A2AR synthTM5 peptide into the nucleus accumbens (Fig. 4a, b). Thus, the response to the A2AR agonist as well as the proportion of D2R in the high affinity state was unaffected.

##### In Situ Proximity Ligation Assay

In the ventral striatum, the intra-accumbal microinjections of the A2AR synthTM5 peptide caused a substantial and significant reduction in the density of A2AR-D2R PLA positive clusters per cell and sampled field in the nucleus accumbens shell and core (Fig. 3c, d). In contrast, in the dorsal striatum, no significant changes were observed in the density of the A2AR-D2R positive PLA clusters (Fig. 4c, d).

## Discussion

The major achievement of the current paper is to provide for the first time evidence at the behavioral and neurochemical levels that one target for the anti-cocaine actions of the A2AR agonist CGS 21680 is the A2AR-D2R heteroreceptor complexes in the nucleus accumbens shell and core. Microinjections of the A2AR synthTM5 peptide, known to be part of the A2AR-D2R receptor interface [21], into the nucleus accumbens produced a complete counteraction of the inhibitory effects of the A2AR agonist on cocaine self-administration. Both the number of active lever presses and cocaine infusions returned to the control levels found in vehicle-treated animals. The neurochemical evidence using the PLA provided significant and clearcut evidence that the A2AR synthTM5 peptide injected into the nucleus accumbens substantially reduced the number of A2AR-D2R heteroreceptor complexes within the nucleus accumbens core and shell without affecting these complexes in the dorsal striatum. Also, the antagonistic A2AR-D2R interactions in the ventral striatum (but not in the dorsal striatum) were significantly counteracted by the A2AR synthTM5 peptide, which is explained by the disappearance of the A2AR-D2R heteromers in these brain structures. The reduction in the affinity of the high affinity D2R agonist binding sites induced by the A2AR agonist was lost.

In the current study, we could also demonstrate that the rat A2AR synthTM5 peptide was highly effective in markedly reducing the BRET^1^ signal in human A2AR-D2R heteromers in HEK293 cell lines. We found in the current paper that the rat A2AR synthTM2 has only weak effects on the BRET^1^ signal of human A2AR-D2R heteromers in high concentrations.

Nevertheless, the specificity of the human A2AR synthTM5 peptide used can still be questioned since its actions on A2AR-D3R heteromers [28] and A2AR-D4R heteromers [29–31] have not yet been tested in cellular models. These A2AR-D2likeR heteroreceptor complexes may exist in the nucleus accumbens in view of the partial codistribution of A2AR and D3R receptors as well as A2AR and D4R receptors in this brain region. Therefore, it is more safe to state that A2AR-D2likeR heteroreceptor complexes were disrupted by the A2AR synthTM5 peptide resulting in the return of cocaine self-administration.

Previous work indicated a role of antagonistic A2AR-D2R interactions in cocaine reward and cocaine seeking [9, 14–16]. Recent evidence coming from our laboratories underlines that cocaine self-administration selectively increases antagonistic allosteric A2AR-D2R interactions in the ventral striatum [17]. In addition, cocaine self-administration specifically increases A2AR-D2R and D2R-sigma1R heteroreceptor complexes in the rat nucleus accumbens shell [18]. Activation of this mechanism may represent a compensatory response to bringing down exaggerated reward induced by cocaine.

Taken together, the results of the current paper give evidence that A2AR-D2Rlike heteroreceptor complexes in the nucleus accumbens with their antagonistic receptor-receptor interactions can be major targets for treatment of cocaine use disorder and help understanding their molecular mechanisms.

## Electronic supplementary material

ESM 1 (PPT 136 kb)
