# Supplementary material for: Disruption of A2AR-D2R Heteroreceptor Complexes After A2AR Transmembrane 5 Peptide Administration Enhances Cocaine Self-Administration in Rats
Source: Mol Neurobiol. 2018 Jan 30;55(8):7038–48. doi: 10.1007/s12035-018-0887-1 (PMC6061166; doi:10.1007/s12035-018-0887-1)
Supplement: Supplementary file 1 — (PPT 136 kb) [file 12035_2018_887_MOESM1_ESM.ppt]

## Slide 1
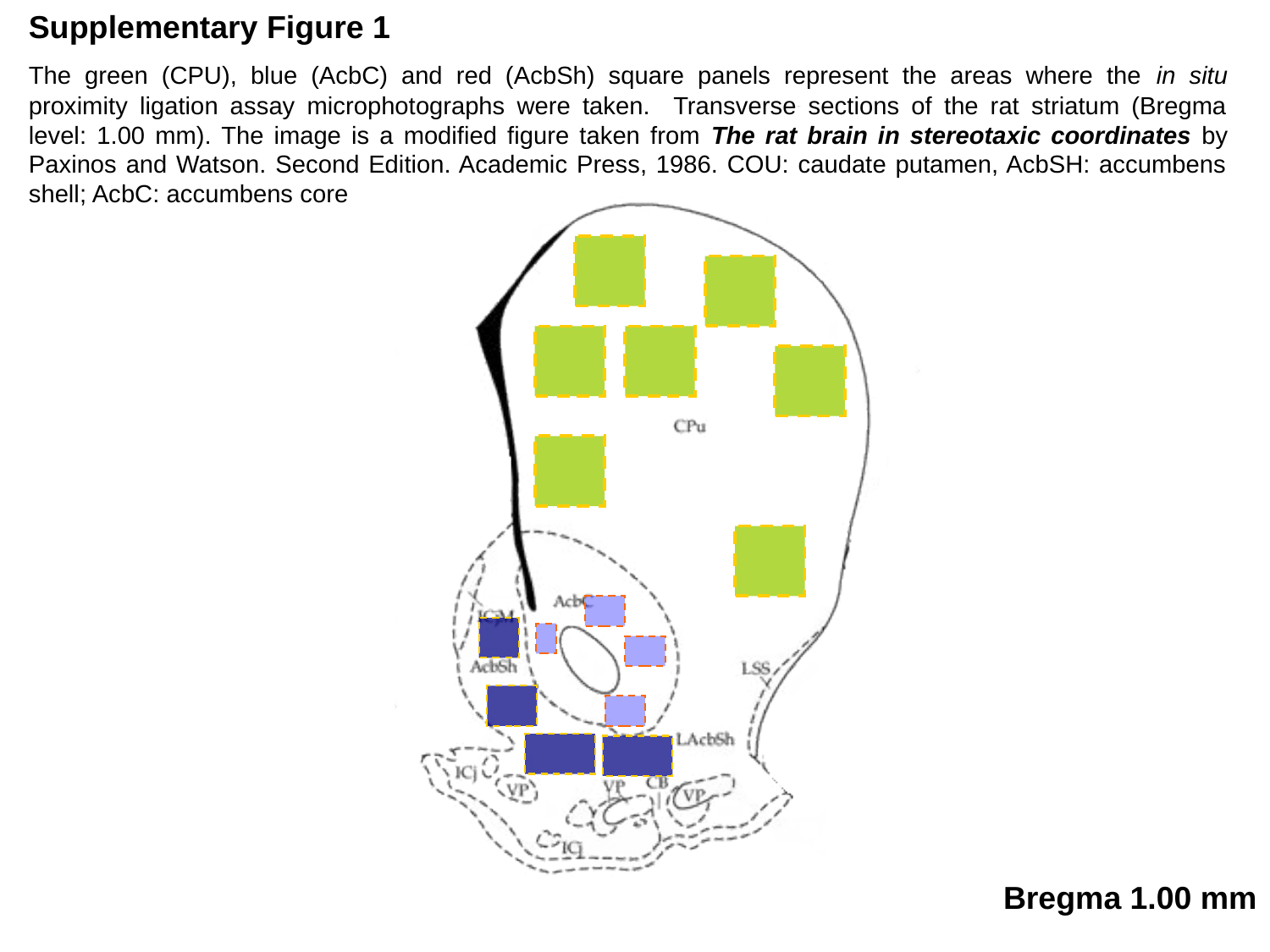

Supplementary Figure 1
The green (CPU), blue (AcbC) and red (AcbSh) square panels represent the areas where the in situ proximity ligation assay microphotographs were taken. Transverse sections of the rat striatum (Bregma level: 1.00 mm). The image is a modified figure taken from The rat brain in stereotaxic coordinates by Paxinos and Watson. Second Edition. Academic Press, 1986. COU: caudate putamen, AcbSH: accumbens shell; AcbC: accumbens core
Bregma 1.00 mm
